# Project YOURLIFE (What Young People Think and Feel about Relationships, Love, Sexuality, and Related Risk Behavior): Cross-sectional and Longitudinal Protocol

**DOI:** 10.3389/fpubh.2016.00028

**Published:** 2016-02-22

**Authors:** Silvia Carlos, Alfonso Osorio, María Calatrava, Cristina Lopez-del Burgo, Miguel Ruiz-Canela, Jokin de Irala

**Affiliations:** ^1^Department of Preventive Medicine and Public Health, University of Navarra, Pamplona, Spain; ^2^Instituto de Investigación Sanitaria de Navarra (IdiSNA), Navarra Institute for Health Research, Pamplona, Spain; ^3^Education of Affectivity and Human Sexuality, Institute for Culture and Society (ICS), University of Navarra, Pamplona, Spain; ^4^School of Education and Psychology, University of Navarra, Pamplona, Spain

**Keywords:** adolescence, sexual behavior, sex education, sexually transmitted infections, sexual health

## Abstract

**Introduction:**

Sexually transmitted infections and unplanned pregnancies affect adolescent sexual health and are serious public health concerns. They result from sexual intercourse in adolescence, which is usually associated with multiple partners, unprotected sex, and condom misuse. This behavior is related to socio-ecological factors that influence lifestyles. The YOURLIFE project aims to find out what young people think and feel about relationships, love, and sexuality, and to assess the associations between these thoughts and attitudes, adolescents’ social factors, and sexual health.

**Materials and equipment:**

An international school-based study with a cross-sectional and optional subsequent longitudinal design. Three online questionnaires designed for adolescents aged 13/14, 15/16, and 17/18, respectively, will be used. A matching coding system will allow longitudinal follow-up when adolescents reply to follow-up surveys. Questionnaires will include questions related to sociodemographic data; information/communication technologies; leisure time; parental supervision; influences of parents/friends; information sources; messages perceived; and sexuality-related knowledge, attitudes, and opinions. The second and third questionnaires for participants aged 15/16 and 17/18 will also contain variables concerning sexual behavior. Schools will be able to use their results to tailor educational approaches targeting the needs of their students. Multivariate analyses will be performed using the larger international dataset.

**Expected impact of the study on public health:**

The YOURLIFE project will collect comprehensive information about the socio-ecological determinants of the sexual risk-taking of schooled adolescents worldwide. Effective preventive programs could be subsequently designed and tailored to the specific determinants of adolescents from different schools and settings, and also, when analyzed globally, to public health professionals.

## Introduction

During adolescence and young adulthood, biological, psychological, and social changes occur, which may be determinants of health or disease in later life (1). For many adolescents, these changes take place without any negative consequences. However, some adolescents adopt risk behaviors that may affect their sexual health (2, 3).

Apart from the psychological effects of adolescent sexual activity, such as depression, regret, and other negative feelings (4), two important outcomes are serious public health concerns: unplanned pregnancies and sexually transmitted infections (STIs) (5–8). It has been estimated that young people aged 15–24 acquire nearly half of all new STIs; that every year, 1 in 20 adolescents contracts a sexual bacterial infection; and that the average age of contagion is decreasing (9). In recent years, there has been a particularly significant increase in STIs among young people, with a high prevalence of human papillomavirus (HPV), *Neisseria gonorrhoeae* or HIV, and the re-emergence of the *Chlamydia trachomatis* infection (10–16).

**Figure 1:**
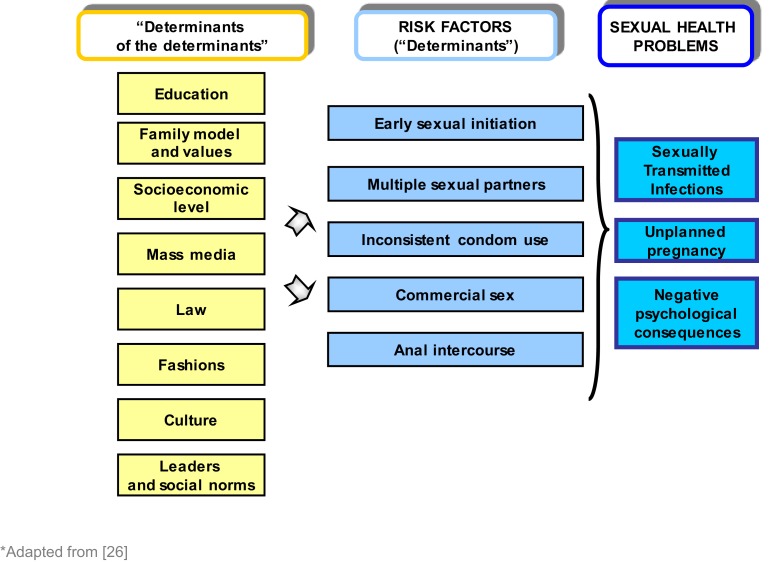
**“The determinants of the determinants”: socio-ecological factors associated with health risk behaviors**.

All these sexual health problems are fueled by sexual intercourse in adolescence, which is in turn associated with having multiple partners, unprotected sex, or condom misuse (17). This risk behavior is, to a large extent, also related to socio-ecological factors, which include the social, cultural, religious, familial, educational, political, and ideological factors that influence lifestyles (Figure 1) (17–26).

Most of these risk factors are modifiable. Therefore, if we manage to influence the socio-cultural context to some extent and, consequently, some adolescents’ sexual behavior, several adolescent sexual health problems could be reduced.

In order to achieve this, effective preventive programs should ideally be based upon reliable information on the social and cultural determinants of their sexual risk-taking. At present, there are various national and international surveillance projects (17, 27–30) that are designed to measure and assess behavioral risk and protective factors among young people. One example of an international surveillance project is the Global School-based Student Health Survey (GSHS). It collects data on health behavior and protective factors from young people aged 13–17, especially from low-income and middle-income countries (29). Another example is the survey on Health Behavior in School-aged Children (HBSC), one of the first international surveys on adolescent health; this survey is restricted to North America and countries in the European Region of the World Health Organization (WHO). It collects data every 4 years on both the social environments and health behavior of 11-, 13-, and 15-year olds (30).

Both international cross-sectional studies have three different types of questionnaires available for the participating countries: a core questionnaire (that addresses the leading causes of morbidity and mortality worldwide), a module with core-expanded questions, and a module with country-specific questions. As far as sexual health is concerned, both the HBSC and the GSHS include questions on possible social determinants of early sexual initiation (i.e., family context, parental supervision, school attendance, bullying at school, characteristics of the young person’s group of friends, leisure time activities, information and communication technologies, and some personal traits). However, in their core questionnaire, they do not inquire about the young people’s knowledge of STI and unplanned pregnancies and their prevention (31, 32). Neither do they include items about affectivity or questions on personal opinions on these topics, the influence of friends’ and parents’ opinions or the messages they receive from the media or from people close to them. In the GSHS core-expanded questions, there are some references to these and other important factors which can influence adolescent behavior, such as young people’s sources of information on sexuality and affectivity (33). Concerning sexual behavior, these surveys inquire about their having had or not having had sexual intercourse and the use of condoms, but significantly, they do not include any core questions about the reasons for having had or for not having had sex, for using or not using condoms, or about feelings regarding their first sexual relationship.

There is therefore ample scope to increase our knowledge of the factors that influence young people and to improve our understanding of their knowledge, beliefs, and opinions concerning sexuality. Obtaining further reliable international data on the determinants of sexual health in adolescents can be useful for the drawing up of general public health guidelines.

One of the reasons why young people are particularly vulnerable is that sex education is often not tailored to their particular needs (34–36). Obtaining further knowledge of the motivations of young people in each particular setting would improve the content of sexual education programs even if they are not necessarily representative of any particular country. More effective educational and preventive communication strategies could be designed and tailored for specific groups of young people.

The primary objective of the YOURLIFE project is to achieve a better understanding on how the socio-ecological context of young people and what they think and feel about relationships, love, and sexuality, may influence their sexual health. The specific objectives of the YOURLIFE project are (1) to study the adolescents’ knowledge, attitudes, and behavior related to sexuality, and thus identify misconceptions as well as risky attitudes and behavior that may put them at an increased risk of acquiring STIs or of unplanned pregnancies; (2) to examine the reasons for having or not having sexual relationships and the circumstances associated with such reasons; and (3) to identify the worldwide social factors associated with particular knowledge, attitudes, and sexual risk behavior using data from schools from different countries and with different sociodemographical characteristics.

The first two objectives will be implemented, both locally in each participating school to achieve specific and targeted educational measures, and also using international data to reach global conclusions on these issues. The third objective will use global regional, national, or international data.

## Materials and Equipment

### Study Design

The YOURLIFE project is an international multipurpose, multicenter, and interdisciplinary survey of adolescents at school. It is not a typical cross-sectional study as it will be constantly opened up to the participation of new schools, and it is not a typical cohort study as follow-up will be shorter than usual, but it will include characteristics of both epidemiological designs. The study will be implemented using different epidemiological strategies:

(a) The use of specific data locally by schools. Three different online questionnaires will be available worldwide for schooled adolescents aged 13/14, 15/16, and 17/18. The surveys will be free of charge and will be available first in Spanish, then in English, and subsequently in other languages as the project expands. Schools will subsequently receive their students’ anonymous results both on pdf documents and on powerpoint slides. This will enable schools to use their results in specific educational programs targeting teachers, parents, and/or students. Any issue picked up by the survey and that warrants the school’s attention as educators of adolescents could be addressed. The survey could also be used by schools to monitor changes in their students once they implement educational programs because the survey can be applied to the same students at different ages.
(b) The use of city, regional, country, or international data in cross-sectional analyses. Data collected from different schools will be classified and examined in the form of cross-sectional analyses. Since we expect a very large number of participating schools worldwide, different analytical strategies of the cross-sectional data will be possible:
  b1. This study is not mainly designed to estimate a representative incidence or prevalence of a given issue. We will therefore not need to use representative samples because we will mainly be looking for associations between a selection of independent and dependent variables. Multivariate analyses will be performed using the whole dataset, and the adjustment of confounders will be possible as we will have a large dataset.
  b2. When descriptive and representative estimations are needed for a given target population, a sampling strategy could be used on the whole dataset in order to select representative data for this specific analysis. For example, if we needed to give a representative estimate from schooled adolescents of a given country, our data from that country would be sampled using specific selection quota on key variables so as to represent schooled adolescents from that country before carrying out the analysis.
(c) The performance of longitudinal follow-ups and analyses of city, regional, country, or international data. Schooled adolescents aged 13/14 can be invited to participate in a subsequent follow-up survey upon reaching the ages of 15/16 or 17/18 and schooled adolescents aged 15/16 can be invited to participate in a subsequent follow-up survey upon reaching 17/18. This prospective analysis of the data will allow improved control of the phenomenon of reverse causality typical in cross-sectional studies and thus allow a better understanding of how some variables in younger participants could be associated with specific outcomes when they are 1 or 2 years older. A set of matching variables will enable the pairing of two questionnaires of the same person at different ages and will preserve the anonymity of the adolescents.

## Participants

### Sample Selection

We will use a convenience sampling method as this will be an international ongoing dynamic and multipurpose study. Schools worldwide will be invited by e-mail and by giving them the link to the website designed to give detailed information to participants (http://www.projectyourlife.com/). This will enable the project to work with a large and heterogeneous number of students. The only limitations will be PC, internet availability, and the language of the questionnaires. The first objective will be to target Spanish-speaking countries, the next will be to proceed in English, and eventually in other languages, which will enable the participation of schools from more countries. In each potential participating country, we will identify lists of schools using official registries and contact them by e-mail. The study participants will consist of schooled adolescents aged 13–18 at the time of recruitment. This convenience sampling method will not necessarily be “representative.” However, one advantage of this sampling scheme is that the quality of the information obtained can be higher if participating schools do so of their own accord and are therefore self-selected with regard to “motivation” (37). Our study does not aim to define the prevalence or incidence of an outcome in the general population or in the population of schooled adolescents. Population or school registries with full coverage would be the most useful tools for this aim. However, the YOURLIFE project intends to study the association between certain attitudes, knowledge, lifestyles, parental education and guidelines given to children, and different health-related outcomes in schooled adolescents. A better quality of data collection is possible because schools taking part voluntarily and motivated to do so can increase the internal validity of results. Once we assume internal validity, we can decide whether the observed associations can be extrapolated to the general population or not. As Rothman points out, the generalization of results in the field of scientific inquiry does not depend on the statistical representativeness of the study population but on the mechanisms underlying the observed association and the biological laws governing the process under study (38, 39). In some situations, selection of a particular group could indeed introduce bias because of the relationship between factors associated with participation in the study, the exposure of interest, and the outcome under study. However, a mere association between a particular factor and attrition does not necessarily induce selection bias (for example, when that factor does not predict the outcome or when other factors with opposite associations compensate for it). In any case, as Hernán et al. suggest, adapting the analysis for these variables could solve the problem (40). In our study, due to the large sample size expected, we will be able to adjust for a large number of confounders that this potential selection bias might introduce.

### Sample Size

Since we will have a dynamic cohort that will be ongoing for some years, we anticipate a sufficiently large sample size to carry out country and international multivariate analyses as well as to adjust for a large number of confounders (41). Our experience in an initial pilot study performed in the region of Navarra in Spain using the online survey for which 80 schools were invited is that approximately 500 students have participated in the survey. Our initial goal is to achieve a minimum participation of around 4,000 students, and we therefore expect to have sufficient statistical power to account for a considerable amount of variables in a given model if we consider the typical multivariate sample estimation rule of “having about 10 respondents for each parameter included in a statistical model” (42). In the past, we have carried out such analyses with samples of around 3,000 students in each country (4, 26, 43–46), and given that this new international cross-sectional and prospective phase of our project will be continuously ongoing, we anticipate a much larger sample size than our initial goal of 4,000. This will enable international and country multivariate analyses, adjusting for a large number of confounders and enabling different subgroup analyses. The study will therefore be similar to typical dynamic multipurpose cohort studies that do not have specific sample size estimations but that count on sufficiently large sample sizes to carry out multivariate analyses and adjust for confounders.

## Stepwise Procedures

### Data Collection

#### The Questionnaire

The YOURLIFE study is based on a self-reporting questionnaire that is specifically designed for adolescents and developed at an easy reading level. It is based on previously validated questionnaires used in several national and international surveys conducted among adolescents (32, 47). The questionnaires have been written first in Spanish, and they will then be translated into the national languages of the respective participating countries.

The surveys will be administered online.

#### Variables

The final version of the questionnaires includes the questions that best suit the research objectives and ages that were targeted. The questionnaires consist of closed questions. Likert scales are employed for most questions, in which students mark the answer that best describes how they value each item.

The questionnaires have six sections in common:

(1) Sociodemographic data;
(2) Leisure time activities;
(3) Tobacco, alcohol, and drug use;
(4) Personality traits and physical or psychological harm;
(5) Family characteristics, relationship, and education;
(6) Knowledge and attitudes concerning affectivity and sexuality.

The questionnaires will be very similar for all the countries, although there may be some differences in the number of items included in the different sections (Table 1). Overall, the number of questions regarding leisure time activities, knowledge and sources of information about sexuality, reasons for deciding to engage in sexual activity or not, and opinions related to sexuality is higher than for the other issues explored.

**Table 1:** **Topics and items assessed in the questionnaires**.

Topics	No. items (13–14 years)	No. items (15–16 years)	No. items (17–18 years)
**Sociodemographic characteristics**	10	8	8
Age, sex, family socioeconomic status, religion (including church assistance and faith relevance), and education			
**Leisure time activities**	44	43	32
Type of leisure time activities and time spent on each activity, use of information and communication technologies, characteristics and quality of group of friends, and money spent			
**Tobacco, alcohol, and drug use**	5	5	5
**Personality traits and physical or psychological harm**	13	13	13
Self-perception of being loved, happiness, feeling free, impulsivity, and tendency to plan things ahead of time			
**Family characteristics, relationship, and education**	97	23	8
Type of family setting and education			
**Information, knowledge, and attitudes concerning affectivity and sexuality**	31	137	144
Source of information, messages, and opinions on affectivity and sexuality, items they talk about with parents and want to learn more about, influence of parents/friends/school/media on different issues, knowledge of risk of unplanned pregnancies and HIV, reasons for having or not having sex, regrets on having or not having had sex, perception of pressure to have sex, information about affectivity and sexuality received in schools, and abortion			
**Practices concerning sexuality**	Not applicable	5	5
Age of first sex, number of sexual partners, and condom use			

The first questionnaire for children aged 13/14 will include more questions concerning family characteristics and educational approaches within the family context and no questions related to sexual activity. The second and third questionnaires for the eldest participants aged 15/16 and 17/18 will contain the items exploring knowledge, attitudes, and practices concerning sexuality. All questionnaires will have a set of similar variables that will enable matching the same person after he/she has responded to follow-up questionnaires and once the prospective follow-up is completed (Table 1).

#### Pilot Study

Pilot studies will be carried out prior to the main study and before implementing each newly translated questionnaire. Adolescents of both sexes and of similar ages to those recruited for the study will be selected to evaluate the comprehension, feasibility, and length of the questionnaire. The questionnaire will have to be implemented within the time available for a normal classroom teaching session.

#### Implementation of the Questionnaire

Schools will be sent an invitation and information on the project by e-mail. Participating schools will receive a protocol with instructions on the survey process. On the survey date, each school will administer the survey during teaching periods.

### Data Analysis

All data will be received at the University of Navarra, where the complete study database will be kept for analysis. Project personnel at the University of Navarra will evaluate all data for consistency and quality. All data from the questionnaires will be automatically entered into a Stata 12.0 database. A descriptive analysis will be carried out first of all to describe the characteristics of the study participants.

Since this is a “multipurpose study,” different hypothesis can be tested using the variables in our questionnaires, and several independent or dependent variables can be used depending on the hypothesis to be tested. Different multivariate statistical models can be adjusted with each using different sets of independent and dependent variables. The main objectives of our study will entail the following analyses: descriptive statistics will be used to study the adolescents’ knowledge, attitudes, and behavior related to sexuality and thus identify misconceptions as well as risky attitudes and behavior that may put them at an increased risk of acquiring STIs or of unplanned pregnancies. Data will be described using means, proportions, and their corresponding 95% confidence intervals when describing continuous and categorical variables, respectively. Significance tests using Student’s *t*-test and chi-squared test will be used when comparing means and proportions, respectively. The reasons for having or for not having sexual relationships will be described using similar statistics. Furthermore, the circumstances associated with such reasons will be analyzed using several multivariate non-conditional logistic regressions in which each reason (or group of reasons) will be used as dependent variable, and the socio-ecological factors, together with other knowledge and educational variables, will be candidates for independent variables and/or confounders.

The social factors worldwide associated with particular knowledge, attitudes, and sexual risk behaviors will be identified using data from schools from different countries and with different sociodemographical characteristics. Several non-conditional logistic regression models will be adjusted using sociodemographical, educational, knowledge, and attitudinal variables as candidate independent variables, and sexual risk behaviors as dependent variables. Independent variables will be picked up from the initial questionnaires and dependent variables from the follow-up questionnaires when longitudinal data from matched questionnaires will be available.

### Ethical Aspects

#### Approval

The overall study design has been approved by the Ethics Committee of the University of Navarra, and each new participating school will be requested to comply with the specific ethical requirements of the project. All the relevant Ethics Committees from the countries participating in the project will have access to the questionnaire before its implementation.

#### Consent

In all cases, after the purpose of the study is clearly explained to the adolescents verbally, written information detailing the project’s objectives and the students’ role in the study will be provided 2 days before the beginning of the implementation. This information will also be given on the first page of the online questionnaire. Consent to participate in the study is thereby established upon acceptance of participation (48). Participants are given the option to withdraw from the study at any time.

Since participating countries may have different requirements concerning parental notification and consent, participating schools will be made aware of the different alternatives they have in order to comply with these requirements; we will explain the framework for the management of parental permission in observational research with adolescents as described by Ruiz-Canela et al. (48). Briefly, the alternatives may include implicit authorization (whether parents are informed or not), the request of an explicit refusal of consent, or the request of an explicit acceptance of consent. Typically, these parental consent alternatives depend on the type of observational research carried out with minors, the risk of harm that participation might entail, and the specific local legislation. They will be described to schools in the “Ethical Considerations” section of the website designed to provide participants with information (http://www.projectyourlife.com/). Before schools receive the verification code to allow participation, they will have to explicitly declare that they have read the ethical considerations section; they will also be required to inform us as to what type of consent they will use for this participation.

#### Confidentiality

The instrument used is a self-administered anonymous questionnaire in which participants are not requested to give their names or any other personal identifying information. Only the school code, school type, and the particular country will be registered in the database. Surveys are designed to ensure this anonymous participation. To increase the respondents’ sense of privacy and promote honest reporting, questionnaires will be administered away from parents, and the protocol establishes that teachers should not move around the computer room while students are answering the questionnaire (49).

The survey data will be kept at the University of Navarra in a secure place. It will not be accessible on the website; access will be restricted to the researchers directly involved in the study. The database linking codes of participating schools and their confidential information (name and address) will be kept separately from the actual survey contents. Data and results enabling the identification of any school in particular will never be published. All analyses published will always include several schools at the same time so as to make the identification of a particular school impossible. If a particular sensitive question is answered positively by 100% of respondents (for example, if 100% of students in a particular classroom respond that they consume alcohol at weekends), the system will not give this information to the school as this would result in breaching confidentiality. However, the school needs to know that they have a problem on this issue, and the system will automatically tell them that “more than 80% of students consume alcohol,” thus enabling educational action, but at the same time respecting confidentiality. Schools will always receive simple descriptive data. For instance, they will not know if smokers consume alcohol, etc.

## Anticipated Results

In this paper, we have described the study profile of the YOURLIFE project, an international study which will monitor what young people think and feel about relationships, love, sexuality, and other possible sexual health-related variables in different schools and countries. In a previous study with a different methodology, we gathered information from approximately 11,000 adolescents from countries worldwide. Some of these countries have not participated in the main international surveys on adolescent health (the GSHS and the survey on HBSC). Based on these previous results concerning the adolescents’ knowledge, attitudes, and behavior related to sexuality and their determinants, we have made all the necessary arrangements for the design of this new project, specifically the online tool and the possibility of implementing an anonymous follow-up of the participants in an efficient manner. We hope that all the data gathered will generate effective preventive programs that will improve adolescents’ sexual health, both locally by taking into account specific school-level circumstances and determinants, and globally by analyzing grouped international data.

The project has several limitations that must be mentioned. First of all, the cross-sectional analyses will usually not lead to the inference of clear causal associations as reverse causality cannot always be ruled out. They may, nevertheless, pinpoint important issues to be further confirmed using longitudinal data. Longitudinal online follow-ups of school cohorts worldwide will enable efficient prospective analyses of causal associations. Another limitation of this study is that the samples of students will not necessarily represent the total population of young people in these age groups. Nevertheless, in-school youth are chosen because one of the implicit objectives of the research is to generate insights on future training methods for this specific group.

Despite these limitations, our study has several strengths. An important strength of the YOURLIFE project is the geographic diversity of its participants, which assures that a wide variation of lifestyles will be represented. The project will include data on countries that are not represented in international surveillance projects on adolescent health. Moreover, the YOURLIFE project is based on a previous project, one of the largest international studies to gather data on the determinants and circumstances of early sexual relationships, with a total approximate sample size of 11,000 adolescents answering a similar questionnaire. Finally, the YOURLIFE project collects data from a wide age range of young people, not only early adolescence but also late adolescence and young adulthood.

## Author Contributions

SC reviewed the literature and wrote the first draft of the manuscript; AO contributed to the questionnaire design and the longitudinal phase design and to the reviewing and writing of further versions of the original draft; MC contributed to the questionnaire design, helped with the protocol design, and writing of the paper; CB contributed to the development of the questionnaire and critically revised the manuscript; MR-C provided advice on ethical aspects and helped write the article; JI, the main researcher for the study, supervised all phases of the study design, worked on the survey development and all the epidemiological and statistical plans, reviewed all versions of the paper, and also contributed by writing subsequent versions of the paper. All authors have read and approved the final version of the manuscript.

## Conflict of Interest Statement

The authors declare that the research was conducted in the absence of any commercial or financial relationships that could be construed as a potential conflict of interest.
