# Supplementary material for: Project YOURLIFE (What Young People Think and Feel about Relationships, Love, Sexuality, and Related Risk Behavior): Cross-sectional and Longitudinal Protocol
Source: Front Public Health. 2016 Feb 22;4:28. doi: 10.3389/fpubh.2016.00028 (PMC4761899; doi:10.3389/fpubh.2016.00028)
Supplement: Supplementary file 1 [file Image_1.PDF]

SURVEY:

YOUTH LIFESTYLES

First, we want to **thank** you for helping us with this study. This is an anonymous international online survey about affectivity, sexuality, and lifestyles. Data from this survey will serve to better inform young people your age about the following: knowledge, attitudes, and needs concerning love and sexuality.

This survey is **ANONYMOUS**. You do not have to write your name on it, which means that no one will be able to identify who answered it. Moreover, only researchers will see your answers; your parents, teachers, and anybody else at your school or institution will not have access to them.

This survey is **VOLUNTARY**. If you do not want to respond to this survey, you can leave the room now or whenever you think appropriate; or you can wait and leave the survey blank until the end. You can choose any of these options. In addition, if you are not comfortable answering a question, don't worry. You can choose to answer with the response, "**I prefer not to answer**," which is available for each of the questions.

It will take about 30 minutes to answer the survey.

We encourage you to participate and thank you for considering our invitation to do so. If you choose to participate in this survey, you will be participating in an international project supported by thousands of young people around the world.

By clicking "Next," you agree to participate in the survey.

Many thanks for your cooperation!

---

1. Answering the survey:
•This survey is not a test. It will not be graded so we encourage you to answer the questions as honestly as possible. These same questions are also used in other countries with different cultures. Therefore, some questions may seem a little strange or irrelevant to your daily life. The questions have several possible answers, but this does not mean that they are all equally healthy or advisable. We state this because in some places, and at certain ages, some people may not be in the circumstances described in those responses. In any case, try to answer those questions as well.
•Each question gives you several response options. **Mark one box for each question**. If you find it difficult to choose between several answers, mark the one closest to what you think or do most of the time.
•In several places, there are questions that refer to your parents. Please apply these questions to your personal circumstances: meaning birth parents, adoptive parents, guardians or the people generally responsible for you.

© ICS

*Study protocol: Carlos S, Osorio A, Calatrava M, Lopez-Del Burgo C, Ruiz-Canela M, de Irala J. Project YOURLIFE (What Young People Think and Feel about Relationships, Love, Sexuality, and Related Risk Behavior): Cross-sectional and Longitudinal Protocol. Front Public Health. 2016;4:28.

2.How old are you?

0□ 10 or younger
1□ 11
2□ 12
3□ 13
4□ 14
5□ 15
6□ 16
7□ 17
8□ 18
9□ 19
10□ 20 or older
11□ I prefer not to answer

3. Are you male or female? 0□ male 1□ female 2□ I prefer not to answer

4. Last school year:

0□ I failed at least one subject
1□ I passed all subjects
2□ I got good grades
3□ I prefer not to answer

5. Please respond to the following questions about new technologies:

	NO	YES	I don't know	I prefer not to answer
Do you have Internet access at home?	0□	1□	2□	3□
Does the Internet network in your home have a filter? (A filter is a program that prohibits access to some web sites) [If you do not have internet access at home mark "NO"]	0□	1□	2□	3□
Do you have a computer (laptop or desktop) in your room?	0□	1□	2□	3□
Do you have a TV in your room?	0□	1□	2□	3□
Do you have a videogame console (PlayStation, PSP, Xbox or similar)?	0□	1□	2□	3□

6. Do you have your own smartphone/tablet etc.?

0□ No → Go to question 9
1□ Yes → Go to the next question
2□ I prefer not to answer → Go to question 9

7. Does your smartphone/Tablet have a data plan?

0□ No
1□ Yes
2□ I prefer not to answer

8. At what age did you have your first personal smartphone/tablet etc.?

0□ 7 or younger
1□ 8
2□ 9
3□ 10
4□ 11
5□ 12
6□ 13
7□ 14
8□ 15
9□ 16 or older
10□ I prefer not to answer

9. Normally, MONDAY THROUGH THURSDAY, how much total time (adding together those 4 days) do you usually spend on the following activities?

	None	Less than 1 hour	Between 1 and 2 hours	Between 2 and 3 hours	Between 3 and 4 hours	Between 4 and 10 hours	More than 10 hours	I prefer not to answer
Study and do homework (outside school hours)	0□	1□	2□	3□	4□	5□	6□	7□
Read books	0□	1□	2□	3□	4□	5□	6□	7□
Read teen magazines	0□	1□	2□	3□	4□	5□	6□	7□
Watch TV	0□	1□	2□	3□	4□	5□	6□	7□
Play video games (console, computer games...)	0□	1□	2□	3□	4□	5□	6□	7□
Work for money	0□	1□	2□	3□	4□	5□	6□	7□

 © ICS

*Study protocol: Carlos S, Osorio A, Calatrava M, Lopez-Del Burgo C, Ruiz-Canela M, de Irala J. Project YOURLIFE (What Young People Think and Feel about Relationships, Love, Sexuality, and Related Risk Behavior): Cross-sectional and Longitudinal Protocol. Front Public Health. 2016;4:28.

10. Please notice. This question is similar to the previous one, but now refers to the **weekend**. Normally, from FRIDAY TO SUNDAY, how much **total** time (adding up those 3 days) do you usually spend on the following activities?

	None	Less than 1 hour	Between 1 and 2 hours	Between 2 and 3 hours	Between 3 and 4 hours	Between 4 and 10 hours	More than 10 hours	I prefer not to answer
Study and do homework (outside school hours)	0☐	1☐	2☐	3☐	4☐	5☐	6☐	7☐
Read books	0☐	1☐	2☐	3☐	4☐	5☐	6☐	7☐
Read teen magazines	0☐	1☐	2☐	3☐	4☐	5☐	6☐	7☐
Watch TV	0☐	1☐	2☐	3☐	4☐	5☐	6☐	7☐
Play video games (console, computer games...)	0☐	1☐	2☐	3☐	4☐	5☐	6☐	7☐
Work for money	0☐	1☐	2☐	3☐	4☐	5☐	6☐	7☐

11. Which of the following types of TV shows do you normally watch (at least once a week)?

	NO	YES	I prefer not to answer
News, sports, cultural programs	0☐	1☐	2☐
Soap operas, youth programming	0☐	1☐	2☐
Reality shows	0☐	1☐	2☐
Variety shows, contests, comedy	0☐	1☐	2☐

12. Please tell us if you agree with the following statements.

	Totally agree	Disagree	Neutral	Agree	Totally agree	I prefer not to respond
I spend time distracted, looking at my Smartphone, Tablet, or computer even if I could be doing more productive things.	0☐	1☐	2☐	3☐	4☐	5☐
I spend more time relating to "virtual friends" than relating to people who are physically around me.	0☐	1☐	2☐	3☐	4☐	5☐
I should be less "dependent" or "connected" to some electronic devices such as the cell phone, tablet or computer.	0☐	1☐	2☐	3☐	4☐	5☐
I spend time watching or replying to mails, chats, tweets….at all times, even interrupting my other activities.	0☐	1☐	2☐	3☐	4☐	5☐
I usually feel uncomfortable when I forget my cell phone or other electronic devices at home, in the car or in any other place, because I feel the need to be connected.	0☐	1☐	2☐	3☐	4☐	5☐
I usually have my cell phone in front of me while I am eating.	0☐	1☐	2☐	3☐	4☐	5☐

13. WE WOULD WANT TO KNOW HOW YOU SPEND YOUR FREE TIME.

14. Do you have friends to count on during difficult moments?

0☐ No
1☐ Yes
2☐ I don´t know
3☐ I prefer not to answer

*Study protocol: Carlos S, Osorio A, Calatrava M, Lopez-Del Burgo C, Ruiz-Canela M, de Irala J. Project YOURLIFE (What Young People Think and Feel about Relationships, Love, Sexuality, and Related Risk Behavior): Cross-sectional and Longitudinal Protocol. Front Public Health. 2016;4:28.

15. Approximately how often do you participate in the following activities?

	Never	A few times a month or less	1–2 days per week	3–5 days per week	Almost or every day	I prefer not to answer
Hang out on the street, in a park, at the beach or in another public place	0☐	1☐	2☐	3☐	4☐	5☐
Sports, mountain hiking, etc.	0☐	1☐	2☐	3☐	4☐	5☐
Volunteering (collaborate with NGOs, charity, etc.)	0☐	1☐	2☐	3☐	4☐	5☐
Attend artistic or educational activities (music, painting, theatre, courses, talks, catechism, etc.)	0☐	1☐	2☐	3☐	4☐	5☐
Go to discos, etc.	0☐	1☐	2☐	3☐	4☐	5☐
Go to shopping centers, game rooms, billiards, football stadium	0☐	1☐	2☐	3☐	4☐	5☐
Hang out inside with your friends without adults present	0☐	1☐	2☐	3☐	4☐	5☐
Do activities with your parents (sports, outings, play board games)	0☐	1☐	2☐	3☐	4☐	5☐
Smoke cigarettes	0☐	1☐	2☐	3☐	4☐	5☐
Consume alcoholic beverages	0☐	1☐	2☐	3☐	4☐	5☐
Consume 5 or more alcoholic drinks a day	0☐	1☐	2☐	3☐	4☐	5☐
Consume pot/marijuana/cannabis	0☐	1☐	2☐	3☐	4☐	5☐
Consume other drugs	0☐	1☐	2☐	3☐	4☐	5☐
Look at erotic or pornographic material	0☐	1☐	2☐	3☐	4☐	5☐
Look at erotic or pornographic material on a mobile phone	0☐	1☐	2☐	3☐	4☐	5☐

17. On average, of the money you usually have (allowance, money you earn working), how much do you usually spend each week?
Answer with the **number** amount, without writing the currency. For example, if you spend about 20 dollars a week, write **<u>20</u>**.

____

20. WE ALSO WANT TO KNOW WHERE YOU GET INFORMATION ON CERTAIN ISSUES

21. When you want to get **information** on issues related to **love and sexuality**, how often do you talk to or consult with the following sources?

	Never	Almost never	Sometimes	Almost always	Always	I prefer not to answer
Your father	0☐	1☐	2☐	3☐	4☐	5☐
Your mother	0☐	1☐	2☐	3☐	4☐	5☐
Your friends	0☐	1☐	2☐	3☐	4☐	5☐
Your boyfriend or girlfriend	0☐	1☐	2☐	3☐	4☐	5☐
A trusted teacher/professor	0☐	1☐	2☐	3☐	4☐	5☐
A trusted counselor (at school, a health center...)	0☐	1☐	2☐	3☐	4☐	5☐
The Internet	0☐	1☐	2☐	3☐	4☐	5☐

© ICS

*Study protocol: Carlos S, Osorio A, Calatrava M, Lopez-Del Burgo C, Ruiz-Canela M, de Irala J. Project YOURLIFE (What Young People Think and Feel about Relationships, Love, Sexuality, and Related Risk Behavior): Cross-sectional and Longitudinal Protocol. Front Public Health. 2016;4:28.

22. Have you talked with your parents (father, mother, or both) about the following issues?

	Not at all	A little	More or less	Fairly often	A lot	I prefer not to answer
Bodily changes in girls (menstruation, breast development, etc.) and/or boys (beard, voice, wet dreams, etc.)	0□	1□	2□	3□	4□	5□
The reproductive system (both male and female)	0□	1□	2□	3□	4□	5□
Pregnancy, when life begins	0□	1□	2□	3□	4□	5□
Abortion	0□	1□	2□	3□	4□	5□
AIDS and other STIs	0□	1□	2□	3□	4□	5□
Condoms and contraceptives	0□	1□	2□	3□	4□	5□
How best to deal with my feelings and affections	0□	1□	2□	3□	4□	5□

24. Please notice. This question is similar to the previous one, but now refers to whether or not you **want to talk more** about these topics.
Indicate whether you would like to talk with your parents more about the following topics:

	NO	YES	I prefer not to answer
Bodily changes in girls (menstruation, breast development, etc.) and/or boys (beard, voice, wet dreams, etc.)	0□	1□	5□
The reproductive system (both male and female)	0□	1□	5□
Pregnancy, when life begins	0□	1□	5□
Abortion	0□	1□	5□
AIDS and other STIs	0□	1□	5□
Condoms and contraceptives	0□	1□	5□
How best to deal with my feelings and affections	0□	1□	5□

26. Please let us know to what extent your school has addressed the following topics:

	Not at all	A little	More or less	Fairly often	A lot	I prefer not to answer
The biological aspects of sexuality (changes in the body, functioning of the reproductive system, pregnancy, AIDS...)	0□	1□	2□	3□	4□	5□
The affective aspects of sexuality (distinguishing between desire, attraction and love and; deal with feelings…)	0□	1□	2□	3□	4□	5□

27. Indicate if you think that your school should speak more, or less, on the following questions.

	They should speak to us much less	They should speak to us less	The level is fine	They should speak to us more	They should speak to us much more	I prefer not to answer
The biological aspects of sexuality	0□	1□	2□	3□	4□	5□
The affective aspects of sexuality	0□	1□	2□	3□	4□	5□

© ICS

*Study protocol: Carlos S, Osorio A, Calatrava M, Lopez-Del Burgo C, Ruiz-Canela M, de Irala J. Project YOURLIFE (What Young People Think and Feel about Relationships, Love, Sexuality, and Related Risk Behavior): Cross-sectional and Longitudinal Protocol. Front Public Health. 2016;4:28.

32. IN WHAT FOLLOWS, WE WANT TO KNOW MORE ABOUT WHAT YOU FEEL AND THINK ABOUT LOVE AND SEXUALITY

In this section, "sexual relationships" refers to full sexual intercourse with penetration.

33. How many people your age do you think have had sex?

	No one or almost no one	Less than half	Half	More than half	Everyone or almost everyone	I prefer not to answer
Young people your age at your school	0☐	1☐	2☐	3☐	4☐	5☐
Young people your age in general	0☐	1☐	2☐	3☐	4☐	5☐

34. What do you think is the risk that the following things occur if you have sex using a condom?

	No risk	Low or medium	High or very high	I don't know	I prefer not to answer
Getting pregnant	0☐	1☐	2☐	3☐	4☐
Contracting HIV	0☐	1☐	2☐	3☐	4☐

58. In your opinion, when does human life begin?
- 0☐ At fertilization (when the egg and sperm unite)
- 1☐ Approximately 15 days after fertilization, when the embryo implants in the uterus
- 2☐ Sometime between embryo implantation in the uterus and birth
- 3☐ At birth
- 4☐ I do not know /I am not sure
- 5☐ I prefer not to answer

59. Do you think a woman has the right to have an abortion?
- 0☐ No
- 1☐ Yes, but only in certain circumstances
- 2☐ Yes, whenever she wants to
- 3☐ I don't know
- 4☐ I prefer not to answer

60. NOW WE WOULD LIKE TO ASK SOME OTHER THINGS ABOUT YOU

61. How many siblings do you have? (Do not include yourself).
- 0 ❑ None
- 1 ❑ 1
- 2 ❑ 2 or more
- 3 ❑ I prefer not to answer

© ICS

*Study protocol: Carlos S, Osorio A, Calatrava M, Lopez-Del Burgo C, Ruiz-Canela M, de Irala J. Project YOURLIFE (What Young People Think and Feel about Relationships, Love, Sexuality, and Related Risk Behavior): Cross-sectional and Longitudinal Protocol. Front Public Health. 2016;4:28.

62. Please indicate with whom you live

	NO	YES	I prefer not to answer
Father	0☐	1☐	2☐
Mother	0☐	1☐	2☐
Another adult (guardian…)	0☐	1☐	2☐
Siblings	0☐	1☐	2☐
Grandparents	0☐	1☐	2☐
Other	0☐	1☐	2☐

63. Your parents' marital status (if either of them are deceased, answer with their marital status when both were alive).

0☐ They never got married (to each other)
1☐ Married
2☐ Separated/divorced; neither has remarried or had a stable partner
3☐ Separated/divorced; at least one has a stable partner or is remarried
4☐ Other
5☐ I prefer not to answer

64. What is the highest educational level that your **father** has completed?

0☐ Uneducated
1☐ Primary Education (or equivalent...): up to 12-14 years old
2☐ Secondary Education: up to 16-18 years old
3☐ Vocational/professional training
4☐ University graduate degree (Diploma, Bachelor's)
5☐ University post-graduate Studies (MA, PhD)
6☐ I do not know
7☐ I prefer not to answer

65. What is the highest educational level that your **mother** has completed?

0☐ Uneducated
1☐ Primary Education (or equivalent...): up to 12-14 years old
2☐ Secondary Education: up to 16-18 years old
3☐ Vocational/professional training
4☐ University graduate degree (Diploma, Bachelor's)
5☐ University post-graduate Studies (MA, PhD)
6☐ I do not know
7☐ I prefer not to answer

66. Please describe how often the following situations apply to your life.

	Never	Almost never	Sometimes	Almost always	Always	I prefer not to answer
I usually have dinner with my parents	0☐	1☐	2☐	3☐	4☐	5☐
I help with housework	0☐	1☐	2☐	3☐	4☐	5☐
I plan my homework	0☐	1☐	2☐	3☐	4☐	5☐
I do things without thinking	0☐	1☐	2☐	3☐	4☐	5☐
I say things without thinking	0☐	1☐	2☐	3☐	4☐	5☐
I usually finish the things/projects that I start	0☐	1☐	2☐	3☐	4☐	5☐
I usually save money	0☐	1☐	2☐	3☐	4☐	5☐

© ICS

*Study protocol: Carlos S, Osorio A, Calatrava M, Lopez-Del Burgo C, Ruiz-Canela M, de Irala J. Project YOURLIFE (What Young People Think and Feel about Relationships, Love, Sexuality, and Related Risk Behavior): Cross-sectional and Longitudinal Protocol. Front Public Health. 2016;4:28.

67. We would like to know to what extent you consider the following relationships satisfactory:

	Not at all	A little	More or less	Somewhat	Very much	Deceased	I prefer not to answer
I consider my parents' relationship (between them) satisfactory	0☐	1☐	2☐	3☐	4☐	5☐	6☐
I consider my relationship with my mother satisfactory	0☐	1☐	2☐	3☐	4☐	5☐	6☐
I consider my relationship with my father satisfactory	0☐	1☐	2☐	3☐	4☐	5☐	6☐

69. Please indicate how often the following situations apply to your life

	Never	Almost never	Sometimes	Almost always	Always	I prefer not to respond
Every now and then, I like admiring a landscape	0☐	1☐	2☐	3☐	4☐	5☐
I like to make myself question about different things in life	0☐	1☐	2☐	3☐	4☐	5☐
Every now and then, I like to be in silence without noise and electronic devices	0☐	1☐	2☐	3☐	4☐	5☐
I generally feel free in my life	0☐	1☐	2☐	3☐	4☐	5☐
I feel loved by others (friends, classmates...)	0☐	1☐	2☐	3☐	4☐	5☐
I am generally happy with the life I lead	0☐	1☐	2☐	3☐	4☐	5☐
I can express my thoughts with my friends without fear	0☐	1☐	2☐	3☐	4☐	5☐
At school I feel accepted by my peers	0☐	1☐	2☐	3☐	4☐	5☐
A student at my school has physically or psychologically harmed or assaulted me.	0☐	1☐	2☐	3☐	4☐	5☐
Someone outside of my school has physically or psychologically harmed or assaulted me	0☐	1☐	2☐	3☐	4☐	5☐

70. What religion do you practice or believe in?

0☐ I do not believe in God/I do not know whether God exists ---------------> Go to question 84
1☐ I believe in God but I don´t have any specific religion
2☐ Catholicism
3☐ Protestantism
4☐ Orthodox religion
5☐ Other Cristian Religions
6☐ Islam
7☐ Hinduism
8☐ Buddhism
8☐ Ethnical religion (Africa, Asia, indigenous America, Europe)
9☐ Chinese folk religion (Taoism, Confucianism)
10☐ Shintoism
11☐ Sikhism
12☐ Judaism
13 ☐ Other
14 ☐ I prefer not to answer

© ICS

*Study protocol: Carlos S, Osorio A, Calatrava M, Lopez-Del Burgo C, Ruiz-Canela M, de Irala J. Project YOURLIFE (What Young People Think and Feel about Relationships, Love, Sexuality, and Related Risk Behavior): Cross-sectional and Longitudinal Protocol. Front Public Health. 2016;4:28.

71. How often do you do the following:

	Never	Almost never	Occasionally in a year	Occasionally in a month	Once a week	More than once a week	I prefer not to answer
Go to church/temple	0□	1□	2□	3□	4□	5□	6□
Pray	0□	1□	2□	3□	4□	5□	5□

72. Do you agree with the following statement? "My faith is an important influence in my life and I am willing to take it into account in my decisions"

	Strongly disagree	Disagree	Neutral	Agree	Strongly agree	I prefer not to answer
Do you agree?	0□	1□	2□	3□	4□	5□

73. PLEASE TELL US SOME THINGS ABOUT YOUR FAMILY

74. You believe your parents:	Never Not at all	Rarely A little	Sometimes Somewhat	Very often A lot	Always Definitely	I prefer not to answer
… Know and understand you well	0□	1□	2□	3□	4□	5□
… Know your friends	0□	1□	2□	3□	4□	5□
… Know your friends' parents	0□	1□	2□	3□	4□	5□
… Talk with your teachers	0□	1□	2□	3□	4□	5□
… Care about your appearance (physical look, clothes, cleanliness)	0□	1□	2□	3□	4□	5□
… Care about you being happy	0□	1□	2□	3□	4□	5□
… Supervise your homework	0□	1□	2□	3□	4□	5□

75. At home, your parents generally:	Never Not at all	Rarely A little	Sometimes Somewhat	Very often A lot	Always Definitely	I prefer not to answer
… Have fun with you	0□	1□	2□	3□	4□	5□
… Share free time with you	0□	1□	2□	3□	4□	5□
… Help you with your studies when you need it	0□	1□	2□	3□	4□	5□
… Speak with you about your interests (hobbies, things you like...)	0□	1□	2□	3□	4□	5□
… You'd like to be like them on important things	0□	1□	2□	3□	4□	5□
… Set an example to you	0□	1□	2□	3□	4□	5□
… Listen to you	0□	1□	2□	3□	4□	5□
… Take your opinions into account when making plans	0□	1□	2□	3□	4□	5□

© ICS

*Study protocol: Carlos S, Osorio A, Calatrava M, Lopez-Del Burgo C, Ruiz-Canela M, de Irala J. Project YOURLIFE (What Young People Think and Feel about Relationships, Love, Sexuality, and Related Risk Behavior): Cross-sectional and Longitudinal Protocol. Front Public Health. 2016;4:28.

76. At home your parents:	Never<br>Not at all	Rarely<br>A little	Sometimes<br>Somewhat	Very often<br>A lot	Always<br>Definitely	I prefer not to answer
… Explain why they reward or punish you	0☐	1☐	2☐	3☐	4☐	5☐
… Make fun of you	0☐	1☐	2☐	3☐	4☐	5☐
… Hit you	0☐	1☐	2☐	3☐	4☐	5☐
… Insult you	0☐	1☐	2☐	3☐	4☐	5☐
… Give you orders, command you, tell you what you have to do	0☐	1☐	2☐	3☐	4☐	5☐
… Explain why you have to do what they command you	0☐	1☐	2☐	3☐	4☐	5☐
… Explain how you have to do what they command you	0☐	1☐	2☐	3☐	4☐	5☐
… Let you do what you feel like	0☐	1☐	2☐	3☐	4☐	5☐
… Require you to follow a schedule at home	0☐	1☐	2☐	3☐	4☐	5☐
… Decide with you what they believe you have to do	0☐	1☐	2☐	3☐	4☐	5☐
… Limit what you spend	0☐	1☐	2☐	3☐	4☐	5☐
… Limit the time you may watch TV	0☐	1☐	2☐	3☐	4☐	5☐
… Let you watch any TV program	0☐	1☐	2☐	3☐	4☐	5☐
… Control your use of the cell phone and internet	0☐	1☐	2☐	3☐	4☐	5☐
… Let you see anything on the internet	0☐	1☐	2☐	3☐	4☐	5☐
… Control the books and magazines you read	0☐	1☐	2☐	3☐	4☐	5☐
… Know what you do during your free time	0☐	1☐	2☐	3☐	4☐	5☐

77. Your parents:	Never<br>Not at all	Rarely<br>A little	Sometimes<br>Somewhat	Very often<br>A lot	Always<br>Definitely	I prefer not to answer
… Speak with you kindly	0☐	1☐	2☐	3☐	4☐	5☐
… Help you when you feel insecure	0☐	1☐	2☐	3☐	4☐	5☐
… Make you feel that they love you, that they accept you as you are	0☐	1☐	2☐	3☐	4☐	5☐
… You feel comforted and supported when you are with them	0☐	1☐	2☐	3☐	4☐	5☐
… You feel they are interested in you	0☐	1☐	2☐	3☐	4☐	5☐
… Have time to talk with you	0☐	1☐	2☐	3☐	4☐	5☐
… Try to be with you and to help you	0☐	1☐	2☐	3☐	4☐	5☐
… Appear indifferent (they don’t care) when you disobey, when you are late, when you don’t study, when you fail, etc.	0☐	1☐	2☐	3☐	4☐	5☐

*Study protocol: Carlos S, Osorio A, Calatrava M, Lopez-Del Burgo C, Ruiz-Canela M, de Irala J. Project YOURLIFE (What Young People Think and Feel about Relationships, Love, Sexuality, and Related Risk Behavior): Cross-sectional and Longitudinal Protocol. Front Public Health. 2016;4:28.

78. You think your parents teach you...	Never<br>Not at all	Rarely<br>A little	Sometimes<br>Somewhat	Very often<br>A lot	Always<br>Definitely	I prefer not<br>to answer
… to be constant with your homework, assignments, etc.	0☐	1☐	2☐	3☐	4☐	5☐
… to see the positive side of things	0☐	1☐	2☐	3☐	4☐	5☐
… not to complain about everything	0☐	1☐	2☐	3☐	4☐	5☐
… to avoid being fussy, picky	0☐	1☐	2☐	3☐	4☐	5☐
… to improve, to achieve your goals	0☐	1☐	2☐	3☐	4☐	5☐
… not to do something only because others do it	0☐	1☐	2☐	3☐	4☐	5☐
… to give your opinion	0☐	1☐	2☐	3☐	4☐	5☐
… to take initiative	0☐	1☐	2☐	3☐	4☐	5☐
… to defend your ideas	0☐	1☐	2☐	3☐	4☐	5☐
… to listen to others people’s ideas	0☐	1☐	2☐	3☐	4☐	5☐
… to avoid certain friends, activities, places	0☐	1☐	2☐	3☐	4☐	5☐

79. You think your parents...	Never<br>Not at all	Rarely<br>A little	Sometimes<br>Somewhat	Very often<br>A lot	Always<br>Definitely	I prefer not<br>to answer
…have cared for you to have friends	0☐	1☐	2☐	3☐	4☐	5☐
…encourage you to invite friends home	0☐	1☐	2☐	3☐	4☐	5☐
…encourage you to relate to different people and groups	0☐	1☐	2☐	3☐	4☐	5☐
…teach you to care about others	0☐	1☐	2☐	3☐	4☐	5☐
…encourage you to volunteer or donate to people who need it	0☐	1☐	2☐	3☐	4☐	5☐
…encourage you to participate in cultural activities (music, painting, etc.)	0☐	1☐	2☐	3☐	4☐	5☐

© ICS

*Study protocol: Carlos S, Osorio A, Calatrava M, Lopez-Del Burgo C, Ruiz-Canela M, de Irala J. Project YOURLIFE (What Young People Think and Feel about Relationships, Love, Sexuality, and Related Risk Behavior): Cross-sectional and Longitudinal Protocol. Front Public Health. 2016;4:28.

80. You think your parents encourage you...	Never<br>Not at all	Rarely<br>A little	Sometimes<br>Somewhat	Very often<br>A lot	Always<br>Definitely	I prefer not to answer
… to socialize with your friends of the opposite sex in a sincere and natural way	0☐	1☐	2☐	3☐	4☐	5☐
… to treat your friends of the opposite sex with respect	0☐	1☐	2☐	3☐	4☐	5☐
… not to have sex until marriage	0☐	1☐	2☐	3☐	4☐	5☐
… not to have sex until there is a commitment	0☐	1☐	2☐	3☐	4☐	5☐
… not to have sex until you are older	0☐	1☐	2☐	3☐	4☐	5☐
… not to have sex until you are ready	0☐	1☐	2☐	3☐	4☐	5☐
… to talk confidently with them (your parents) when you have concerns or difficulties with your friends of the opposite sex	0☐	1☐	2☐	3☐	4☐	5☐
… to distinguish between attraction, infatuation, and love	0☐	1☐	2☐	3☐	4☐	5☐
… to focus on more than just the physical aspect when valuing people	0☐	1☐	2☐	3☐	4☐	5☐

81. Your parents taught you that...	Never<br>Not at all	Rarely<br>A little	Sometimes<br>Somewhat	Very often<br>A lot	Always<br>Definitely	I prefer not to answer
… boys and girls have different ways of being	0☐	1☐	2☐	3☐	4☐	5☐
… boys and girls tend to express their emotions differently	0☐	1☐	2☐	3☐	4☐	5☐
… boys and girls tend to interpret signs of affection differently	0☐	1☐	2☐	3☐	4☐	5☐
… boys are superior to girls	0☐	1☐	2☐	3☐	4☐	5☐
… girls are superior to boys	0☐	1☐	2☐	3☐	4☐	5☐
… a boy and a girl can perform any work or task just as well	0☐	1☐	2☐	3☐	4☐	5☐

82. You believe that...	Never<br>Not at all	Rarely<br>A little	Sometimes<br>Somewhat	Very often<br>A lot	Always<br>Definitely	I prefer not to answer
…your father explained the development of sexuality clearly to you	0☐	1☐	2☐	3☐	4☐	5☐
… your mother explained the development of sexuality clearly to you	0☐	1☐	2☐	3☐	4☐	5☐

 © ICS

*Study protocol: Carlos S, Osorio A, Calatrava M, Lopez-Del Burgo C, Ruiz-Canela M, de Irala J. Project YOURLIFE (What Young People Think and Feel about Relationships, Love, Sexuality, and Related Risk Behavior): Cross-sectional and Longitudinal Protocol. Front Public Health. 2016;4:28.

83. Your parents encourage you...	Never Not at all	Rarely A little	Sometimes Somewhat	Very often A lot	Always Definitely	I prefer not to answer
… not to be curious and to respect the privacy of others	0☐	1☐	2☐	3☐	4☐	5☐
… not to share your problems and feelings with people who are not trustworthy	0☐	1☐	2☐	3☐	4☐	5☐
… to dress appropriately so that you don't make others feel uncomfortable	0☐	1☐	2☐	3☐	4☐	5☐
… to avoid seeing pictures or listening to songs with sexual content	0☐	1☐	2☐	3☐	4☐	5☐
… that the privacy of your body is important	0☐	1☐	2☐	3☐	4☐	5☐
… to take care of your physical appearance	0☐	1☐	2☐	3☐	4☐	5☐
… not to be obsessed about your physical appearance	0☐	1☐	2☐	3☐	4☐	5☐
… to avoid lying and pretending, in chats or social networks	0☐	1☐	2☐	3☐	4☐	5☐
… not to give personal information (yours, your families or your friends') to other people over the Internet	0☐	1☐	2☐	3☐	4☐	5☐
… not to take pictures, record conversations, or post other people's stuff online without permission	0☐	1☐	2☐	3☐	4☐	5☐
… not to talk in public about things that you know about friends or their families	0☐	1☐	2☐	3☐	4☐	5☐

84. Which is the biggest pet you had during your childhood, before 7th grade?

0☐ None
1☐ Spider or insect
2☐ Bunny
3☐ Cat
4☐ Hamster or Guinea pig
5☐ Bird
6☐ Dog
7☐ Fish, small turtle
8☐ Lizard, iguana, other reptile
9☐ Other
10☐ I prefer not to answer

© ICS

85. Considering all of the places you went on holiday/vacation during your childhood, before 7th grade, from which one do you have best memories?

0☐ None
1☐ Countryside, mountains, village
2☐ Camping
3☐ Beach
4☐ Amusement/theme park/water park
5☐ Other
6☐ I prefer not to answer

86. Which was your least favorite food during your childhood, before 7th grade?

0☐ None
1☐ Cauliflower or Broccoli or Brussel sprouts
2☐ Vegetables in general or fruits
3☐ Onion
4☐ Brain, liver, kidneys
5☐ Fish
6☐ Cheese
7☐ Mushrooms
8☐ Tomatoes or green pees
9☐ Other
10☐ I prefer not to answer

87. Which of the following sports did you like the most in the 7th grade? If it is not on the list, please choose your next favorite sport.

0☐ Basketball
1☐ Handball
2☐ Horseback riding
3☐ Skiing or Snowboarding
4☐ Gymnastics, dance or track and field
5☐ Golf
6☐ Biking
7☐ Swimming or water polo
8☐ Tennis, squash, any other racket sport
9☐ Volleyball or baseball
10☐ Skating
11☐ I don't like any of these sports
12☐ I prefer not to answer

© ICS

*Study protocol: Carlos S, Osorio A, Calatrava M, Lopez-Del Burgo C, Ruiz-Canela M, de Irala J. Project YOURLIFE (What Young People Think and Feel about Relationships, Love, Sexuality, and Related Risk Behavior): Cross-sectional and Longitudinal Protocol. Front Public Health. 2016;4:28.

88. Which was your favorite color in 7th grade?

0☐ None
1☐ White
2☐ Gray
3☐ Black
4☐ Brown
5☐ Red
6☐ Pink
7☐ Orange
8☐ Yellow
9☐ Green
10☐ Blue
11☐ Violet
12☐ Other
13☐ I prefer not to answer

89. Which was your favorite number in 7th grade?

0☐ None
1☐ 1
2☐ 2
3☐ 3
4☐ 4
5☐ 5
6☐ 6
7☐ 7
8☐ 8
9☐ 9
10☐ 10
11☐ 11
12☐ 12
13☐ 13
14☐ 14
15☐ 15
16☐ 16
17☐ 17
18☐ 18
19☐ 19
20☐ 20
21☐ Other
22☐ I prefer not to answer

90. WE THANK YOU FOR BEING WILLING TO SHARE THIS INFORMATION WITH US.

Lastly, we want you to know that even if we asked some questions about certain health-related behaviors, this does not imply that we expected you to have those behaviors. These questions are routinely asked as requirements of the study.

You will finish the survey by clicking this button.

© ICS

*Study protocol: Carlos S, Osorio A, Calatrava M, Lopez-Del Burgo C, Ruiz-Canela M, de Irala J. Project YOURLIFE (What Young People Think and Feel about Relationships, Love, Sexuality, and Related Risk Behavior): Cross-sectional and Longitudinal Protocol. Front Public Health. 2016;4:28.
