# Supplementary material for: Project YOURLIFE (What Young People Think and Feel about Relationships, Love, Sexuality, and Related Risk Behavior): Cross-sectional and Longitudinal Protocol
Source: Front Public Health. 2016 Feb 22;4:28. doi: 10.3389/fpubh.2016.00028 (PMC4761899; doi:10.3389/fpubh.2016.00028)
Supplement: Supplementary file 2 [file Image_2.PDF]

SURVEY:

YOUTH LIFESTYLES

First, we want to **thank** you for helping us with this study. This is an anonymous international online survey about affectivity, sexuality, and lifestyles. Data from this survey will serve to better inform young people your age about the following: knowledge, attitudes, needs concerning love, and sexuality.

This survey is **ANONYMOUS**. You do not have to write your name on it, which means that no one will be able to identify who answered it. Moreover, only researchers will see your answers; your parents, teachers, and anybody else at your school will not have access to them.

This survey is **VOLUNTARY**. If you do not want to respond to this survey, you can leave the room now or whenever you think appropriate; or you can wait and leave the survey blank until the end. You can choose any of these options. In addition, if you are not comfortable answering a question, don't worry. You can choose to answer with the response, "**I prefer not to answer**," which is available for each of the questions.

It will take about 30 minutes to answer the survey.

We encourage you to participate and thank you for considering our invitation to do so. If you choose to participate in this survey, you will participate in an international project supported by thousands of young people around the world.

By clicking "Next," you agree to participate in the survey.

Many thanks for your cooperation!

---

1. Answering the survey:
•This survey is **not a test**. It will not be graded so we encourage you to answer the questions as honestly as possible. These same questions are also used in other countries with different cultures. Therefore, some questions may seem a little strange or irrelevant to your daily life. The questions have several possible answers, but this does not mean that they are all equally healthy or advisable. We state this because in some places, and at certain ages, some people may not be in the circumstances described in those responses. In any case, try to answer those questions as well.
•Each question gives you several response options. **Mark one box for each question**. If you find it difficult to choose between several answers, mark the one closest to what you think or do most of the time.
•In several places, there are questions that refer to your **parents**. Please apply these questions to your personal circumstances: meaning birth parents, adoptive parents, guardians or the people generally responsible for you.

---

*Study protocol: Carlos S, Osorio A, Calatrava M, Lopez-Del Burgo C, Ruiz-Canela M, de Irala J. Project YOURLIFE (What Young People Think and Feel about Relationships, Love, Sexuality, and Related Risk Behavior): Cross-sectional and Longitudinal Protocol. Front Public Health. 2016;4:28.

2.How old are you?

0☐ 10 or younger	3☐ 13	6☐ 16	9☐ 19
1☐ 11	4☐ 14	7☐ 17	10☐ 20 or older
2☐ 12	5☐ 15	8☐ 18	11☐ I prefer not to answer

3. Are you male or female? 0☐ male 1☐ female 2☐ I prefer not to answer

4. Last school year:
- 0☐ I failed at least one subject
- 1☐ I passed all subjects
- 2☐ I got good grades
- 3☐ I prefer not to answer

5. Please respond to questions about new technologies:

	NO	YES	I don't know	I prefer not to answer
Do you have Internet access at home?	0☐	1☐	2☐	3☐
Does the Internet network in your home have a filter? (A filter is a program that prohibits access to some web sites) [If you do not have internet access at home mark "NO"]	0☐	1☐	2☐	3☐
Do you have a computer (or laptop) in your room?	0☐	1☐	2☐	3☐
Do you have a TV in your room?	0☐	1☐	2☐	3☐
Do you have a videogame console (PlayStation, PSP, Xbox or similar)?	0☐	1☐	2☐	3☐

6. Do you have your own smartphone/tablet etc.?
- 0☐ No → Go to question 9
- 1☐ Yes → Go to the next question
- 2☐ I prefer not to answer → Go to question 9

7. Does your smartphone/Tablet have a data plan?
- 0☐ No
- 1☐ Yes
- 2☐ I prefer not to answer

9 Normally, MONDAY THROUGH THURSDAY, how much **total** time (adding together those 4 days) do you usually spend on the following activities?

	None	Less than 1 hour	Between 1 and 2 hours	Between 2 and 3 hours	Between 3 and 4 hours	Between 4 and 10 hours	More than 10 hours	I prefer not to answer
Study and do homework (outside school hours)	0☐	1☐	2☐	3☐	4☐	5☐	6☐	7☐
Read books	0☐	1☐	2☐	3☐	4☐	5☐	6☐	7☐
Read teen magazines	0☐	1☐	2☐	3☐	4☐	5☐	6☐	7☐
Watch TV	0☐	1☐	2☐	3☐	4☐	5☐	6☐	7☐
Play video games (console, computer games...)	0☐	1☐	2☐	3☐	4☐	5☐	6☐	7☐
Work for money	0☐	1☐	2☐	3☐	4☐	5☐	6☐	7☐

10. Please notice. This question is similar to the previous one, but now refers to the **weekend**. Normally, from FRIDAY TO SUNDAY, how much **total** time (adding up those 3 days) do you usually spend on the following activities?

	None	Less than 1 hour	Between 1 and 2 hours	Between 2 and 3 hours	Between 3 and 4 hours	Between 4 and 10 hours	More than 10 hours	I prefer not to answer
Study and do homework (outside school hours)	0☐	1☐	2☐	3☐	4☐	5☐	6☐	7☐
Read books	0☐	1☐	2☐	3☐	4☐	5☐	6☐	7☐
Read teen magazines	0☐	1☐	2☐	3☐	4☐	5☐	6☐	7☐
Watch TV	0☐	1☐	2☐	3☐	4☐	5☐	6☐	7☐
Play video games (console, computer games...)	0☐	1☐	2☐	3☐	4☐	5☐	6☐	7☐
Work for money	0☐	1☐	2☐	3☐	4☐	5☐	6☐	7☐

11. Which of the following types of TV shows do you normally watch (at least once a week)?

	NO	YES	I prefer not to answer
News, sports, cultural programs	0☐	1☐	2☐
Soap operas, youth programming	0☐	1☐	2☐
Reality shows	0☐	1☐	2☐
Variety shows, contests, comedy	0☐	1☐	2☐

12. Please indicate to what extent you agree with the following situations.

	Totally Disagree	Disagree	Neutral	Agree	Totally Agree	I prefer not to answer
I spend time absently looking at my Smartphone, Tablet, or computer even when I could be doing more productive things.	0☐	1☐	2☐	3☐	4☐	5☐
I spend more time interacting with "virtual friends" than with people who are physically with me.	0☐	1☐	2☐	3☐	4☐	5☐
I should be less "addicted" or "connected" to some electronic devices such as my cell phone, Tablet and/or computer.	0☐	1☐	2☐	3☐	4☐	5☐
At all times, I look at and answer emails, chats, and tweets, even when doing so interrupts other activities.	0☐	1☐	2☐	3☐	4☐	5☐
I usually feel uncomfortable when I carelessly leave my cell phone or another electronic device at home, in the car, or in another place because I feel the need to be connected.	0☐	1☐	2☐	3☐	4☐	5☐
While I eat, I have my cell phone in front of me.	0☐	1☐	2☐	3☐	4☐	5☐

13. WE WANT TO KNOW HOW YOU SPEND YOUR FREE TIME.

14. Do you have friends to count on during difficult moments?

0□ No
1□ Yes
2□ I don´t know
3□ I prefer not to answer

15. Approximately how often do you participate in the following activities?

	Never	A few times a month or less	1–2 days per week	3–5 days per week	Almost or every day	I prefer not to answer
Hang out on the street, in a park, at the beach or in another public place	0□	1□	2□	3□	4□	5□
Sports, mountain hiking, etc.	0□	1□	2□	3□	4□	5□
Volunteering (collaborate with NGOs, charity, etc.)	0□	1□	2□	3□	4□	5□
Attend artistic or educational activities (music, painting, theatre, courses, talks, catechism, etc.)	0□	1□	2□	3□	4□	5□
Go to discos, etc.	0□	1□	2□	3□	4□	5□
Go to shopping centers, game rooms, billiards, football stadium	0□	1□	2□	3□	4□	5□
Hang out inside with your friends without adults present	0□	1□	2□	3□	4□	5□
Do activities with your parents (sports, outings, play board games)	0□	1□	2□	3□	4□	5□
Smoke cigarettes	0□	1□	2□	3□	4□	5□
Consume alcoholic beverages	0□	1□	2□	3□	4□	5□
Consume 5 or more alcoholic drinks a day	0□	1□	2□	3□	4□	5□
Consume pot/marijuana/cannabis	0□	1□	2□	3□	4□	5□
Consume other drugs	0□	1□	2□	3□	4□	5□
Look at erotic or pornographic material	0□	1□	2□	3□	4□	5□
Look at erotic or pornographic material on a mobile phone	0□	1□	2□	3□	4□	5□

16. Do you believe your parents or your legal guardians…

	Never	Rarely	Sometimes	Almost always	Always	I prefer not to respond
… supervise your homework	0□	1□	2□	3□	4□	5□
… know what you do during your free time	0□	1□	2□	3□	4□	5□
… Require you to follow a schedule at home	0□	1□	2□	3□	4□	5□
... limit what you spend	0□	1□	2□	3□	4□	5□
…limit the time you spend watching TV.	0□	1□	2□	3□	4□	5□
… allow you to see any program on TV.	0□	1□	2□	3□	4□	5□
… control your use of cell phone or the internet	0□	1□	2□	3□	4□	5□
… allow you to see anything on the internet	0□	1□	2□	3□	4□	5□
… control your books and magazines	0□	1□	2□	3□	4□	5□

17. On average, of the money you usually have (allowance, money you earn working), how much do you usually spend each week?
Answer with the **number** amount, without writing the currency. For example, if you spend about 20 dollars a week, write **20**.

____

18. Is what **YOUR PARENTS** think on the following issues important **TO YOU**?

	Not important	A little important	More or less important	Important	Very important	I prefer not to answer
…smoking, alcohol, or drug consumption	0☐	1☐	2☐	3☐	4☐	5☐
…what you do during your free time	0☐	1☐	2☐	3☐	4☐	5☐
…issues concerning love and sexuality	0☐	1☐	2☐	3☐	4☐	5☐

19. Please notice. This question is similar to the previous one, but now it refers to the importance **YOU GIVE** to what **YOUR FRIENDS** think.

Is what **YOUR FIRENDS THINK** on the following issues important **TO YOU**?

	Not important	A little important	More or less important	Important	Very important	I prefer not to answer
…smoking, alcohol, or drug consumption	0☐	1☐	2☐	3☐	4☐	5☐
…what you do during your free time	0☐	1☐	2☐	3☐	4☐	5☐
…issues concerning love and sexuality	0☐	1☐	2☐	3☐	4☐	5☐

20. WE ALSO WANT TO KNOW WHERE YOU GET INFORMATION ON CERTAIN ISSUES

21. When you want to get **information** on issues related to **love and sexuality**, how often do you talk to or consult with the following sources?

	Never	Almost never	Sometimes	Almost always	Always	I prefer not to answer
Your father	0☐	1☐	2☐	3☐	4☐	5☐
Your mother	0☐	1☐	2☐	3☐	4☐	5☐
Your friends	0☐	1☐	2☐	3☐	4☐	5☐
Your boyfriend or girlfriend	0☐	1☐	2☐	3☐	4☐	5☐
A trusted teacher/professor	0☐	1☐	2☐	3☐	4☐	5☐
Youth Counseling (at school, a health center...)	0☐	1☐	2☐	3☐	4☐	5☐
The Internet	0☐	1☐	2☐	3☐	4☐	5☐

23. Did you talk to your parents about the following issues?

	Never	Rarely	Sometimes	Often	A lot	I prefer not to answer
How to know when I will be prepared to have sexual relationships	0☐	1☐	2☐	3☐	4☐	5☐
If I do not want to have sex, how to say it to the person I am dating (boyfriend/girlfriend)	0☐	1☐	2☐	3☐	4☐	5☐
How to know whether the person I am dating (boyfriend/girlfriend) is the right person with whom to build a future together	0☐	1☐	2☐	3☐	4☐	5☐
How best to deal with my feelings and affections	0☐	1☐	2☐	3☐	4☐	5☐
How best to deal with my sexual urges	0☐	1☐	2☐	3☐	4☐	5☐
How to distinguish between sexual attraction, infatuation, and love.	0☐	1☐	2☐	3☐	4☐	5☐

25. Please notice. This question is similar to the previous one, but now refers to whether or not you **want to talk more** about these topics.
Indicate whether you would like to talk with your parents more about the following topics:

	NO	YES	I prefer not to answer
How to know when I will be prepared to have sexual relationships	0☐	1☐	5☐
If I do not want to have sex, how to say it to the person I am dating (boyfriend/girlfriend)	0☐	1☐	5☐
How to know whether the person I am dating (boyfriend/girlfriend) is the right person with whom to build a future together	0☐	1☐	5☐
How best to deal with my feelings and affections	0☐	1☐	5☐
How best to deal with my sexual urges	0☐	1☐	5☐
How to distinguish between sexual attraction, infatuation, and love.	0☐	1☐	5☐

26. Please let us know to what extent your school has approached the following topics:

	Not at all	A little	More or less	Fairly often	A lot	I prefer not to answer
The biological aspects of sexuality (changes in the body, functioning of the reproductive system, pregnancy, AIDS...)	0☐	1☐	2☐	3☐	4☐	5☐
The affective aspects of sexuality (distinguishing between desire, attraction and love and; deal with feelings, sex drive, how to know when I'll be ready for sex...)	0☐	1☐	2☐	3☐	4☐	5☐

27. Indicate if you think that your school should speak more, or less, on the following questions.

	They should speak to us much less	They should speak to us less	The level is fine	They should speak to us more	They should speak to us much more	I prefer not to answer
The biological aspects of sexuality	0☐	1☐	2☐	3☐	4☐	5☐
The affective aspects of sexuality	0☐	1☐	2☐	3☐	4☐	5☐

28. Do you believe **YOUR PARENTS** convey the following messages to you in favor of…?

	NO	YES	I prefer not to answer
… if you get married, your marriage should last your whole life	0☐	1☐	2☐
… waiting until marriage before having sexual relationships	0☐	1☐	2☐
… having sexual relationships because it is risk free (“it´s ok to have sex”)	0☐	1☐	2☐
… having sexual relationships to have fun, even if there is no commitment involved	0☐	1☐	2☐
… using condoms if you decide to have sex	0☐	1☐	2☐

29. Do you believe **YOUR SCHOOL** conveys the following messages to you in favor of…?

	NO	YES	I prefer not to answer
…waiting until marriage before having sexual relationships	0☐	1☐	2☐
…having sexual relationships because it is risk free (“it´s ok to have sex”)	0☐	1☐	2☐
…having sexual relationships to have fun, even if there is no commitment involved	0☐	1☐	2☐
…using condoms if you decide to have sex	0☐	1☐	2☐

30. Do you believe **YOUR FRIENDS** convey the following messages to you in favor of…?

	NO	YES	I prefer not to answer
…waiting until marriage before having sexual relationships	0☐	1☐	2☐
…having sexual relationships because it is risk free (“it´s ok to have sex”)	0☐	1☐	2☐
…having sexual relationships to have fun, even if there is no commitment involved	0☐	1☐	2☐
…using condoms if you decide to have sex	0☐	1☐	2☐

31. Do you believe **THE MEDIA** convey the following messages to you in favor of…?

	NO	YES	I prefer not to answer
… waiting until marriage before having sexual relationships	0☐	1☐	2☐
…having sexual relationships because it is risk free (“it´s ok to have sex”)	0☐	1☐	2☐
…having sexual relationships to have fun, even if there is no commitment involved	0☐	1☐	2☐
…using condoms if you decide to have sex	0☐	1☐	2☐

32. IN WHAT FOLLOWS, WE WANT TO KNOW MORE ABOUT WHAT YOU FEEL AND THINK ABOUT LOVE AND SEXUALITY

In this section, "sexual relations" refers to <u>full sexual intercourse with penetration</u>.

33. How many people your age do you think have had sex?

	No one or almost no one	Less than half	Half	More than half	Everyone or almost everyone	I prefer not to answer
Young people your age at your school/high school	0☐	1☐	2☐	3☐	4☐	5☐
Young people your age in general	0☐	1☐	2☐	3☐	4☐	5☐

©ICS
*Study protocol: Carlos S, Osorio A, Calatrava M, Lopez-Del Burgo C, Ruiz-Canela M, de Irala J. Project YOURLIFE (What Young People Think and Feel about Relationships, Love, Sexuality, and Related Risk Behavior): Cross-sectional and Longitudinal Protocol. Front Public Health. 2016;4:28.

34. What do you think is the risk that the following things occur if you have sex using a **condom**?

	No risk	Low or medium	High or very high	I don't know	I prefer not to answer
Getting pregnant	0☐	1☐	2☐	3☐	4☐
Contracting HIV	0☐	1☐	2☐	3☐	4☐

35. **Let us assume one always uses condoms** while having sex. Please estimate the risk of contracting a sexually transmitted infection in the following situations:

	0 No risk	1	2 Average	3	4 Very high	I prefer not to answer
Having only one lifetime sexual partner	0☐	1☐	2☐	3☐	4☐	5☐
Having 2 or more lifetime sexual partners (not simultaneously, i.e. having at each period only one stable partner to whom one is faithful during that period)	0☐	1☐	2☐	3☐	4☐	5☐
Having 2 or more concurrent sexual partners (during the same period).	0☐	1☐	2☐	3☐	4☐	5☐

36. Do you consider the following aspects important when choosing a partner?

	Not important	A little important	More or less important	Important	Essential	I prefer not to answer
That I like him/her physically (handsome/beautiful, has a good body…)	0☐	1☐	2☐	3☐	4☐	5☐
That he/she has money, a motorcycle, a car…	0☐	1☐	2☐	3☐	4☐	5☐
That he/she is a good person, solidary, loyal, faithful	0☐	1☐	2☐	3☐	4☐	5☐
That he/she is intelligent	0☐	1☐	2☐	3☐	4☐	5☐
That he/she is sensitive	0☐	1☐	2☐	3☐	4☐	5☐
That he/she has a sense of humor	0☐	1☐	2☐	3☐	4☐	5☐
That he/she is responsible, a good student	0☐	1☐	2☐	3☐	4☐	5☐
That he/she is willing to wait until marriage before having sex.	0☐	1☐	2☐	3☐	4☐	5☐
That we both have the common hobbies or interests	0☐	1☐	2☐	3☐	4☐	5☐
That we both have similar principles about important issues (values, faith…)	0☐	1☐	2☐	3☐	4☐	5☐
That I can see that I could count on her/him in my difficult times.	0☐	1☐	2☐	3☐	4☐	5☐

©ICS
*Study protocol: Carlos S, Osorio A, Calatrava M, Lopez-Del Burgo C, Ruiz-Canela M, de Irala J. Project YOURLIFE (What Young People Think and Feel about Relationships, Love, Sexuality, and Related Risk Behavior): Cross-sectional and Longitudinal Protocol. Front Public Health. 2016;4:28.

37. Please notice. This question is similar to the previous one but now we are referring to priorities for the sex opposite to yours.
Do you believe young people from the opposite sex consider the following aspects important when choosing a partner?

	Not important	A little important	More or less important	Important	Essential	I prefer not to answer
That one likes him/her physically (handsome/beautiful, has a good body…)	0☐	1☐	2☐	3☐	4☐	5☐
That he/she has money, a motorcycle, a car…	0☐	1☐	2☐	3☐	4☐	5☐
That he/she is a good person, solidary, loyal, faithful	0☐	1☐	2☐	3☐	4☐	5☐
That he/she is intelligent	0☐	1☐	2☐	3☐	4☐	5☐
That he/she is sensitive	0☐	1☐	2☐	3☐	4☐	5☐
That he/she has a sense of humor	0☐	1☐	2☐	3☐	4☐	5☐
That he/she is responsible, a good student	0☐	1☐	2☐	3☐	4☐	5☐
That he/she is willing to wait until marriage before having sex.	0☐	1☐	2☐	3☐	4☐	5☐
That both have common hobbies or interests	0☐	1☐	2☐	3☐	4☐	5☐
That both have similar principles about important issues (values, faith…)	0☐	1☐	2☐	3☐	4☐	5☐
That one can see he/she could count on her/him in difficult times.	0☐	1☐	2☐	3☐	4☐	5☐

38. Do you agree with the following statements?

	Strongly disagree	Disagree	Undecided	Agree	Strongly agree	I prefer not to answer
Paternity/maternity are important aspects in the lives of married persons	0☐	1☐	2☐	3☐	4☐	5☐
In the future I would like to have children	0☐	1☐	2☐	3☐	4☐	5☐
I agree that a young couple can sexually arouse each other independently of them having complete sexual relationships or not	0☐	1☐	2☐	3☐	4☐	5☐
A love that lasts forever is possible	0☐	1☐	2☐	3☐	4☐	5☐
I would like to find a love that lasts forever	0☐	1☐	2☐	3☐	4☐	5☐
Having sex before marriage increases the likelihood that your marriage goes well	0☐	1☐	2☐	3☐	4☐	5☐
For love to last it is necessary that both be ready to make sacrifices for each other.	0☐	1☐	2☐	3☐	4☐	5☐
Male virginity is important	0☐	1☐	2☐	3☐	4☐	5☐
Female virginity is important	0☐	1☐	2☐	3☐	4☐	5☐

©ICS
*Study protocol: Carlos S, Osorio A, Calatrava M, Lopez-Del Burgo C, Ruiz-Canela M, de Irala J. Project YOURLIFE (What Young People Think and Feel about Relationships, Love, Sexuality, and Related Risk Behavior): Cross-sectional and Longitudinal Protocol. Front Public Health. 2016;4:28.

39. Do you agree with the following statements?

	Strongly disagree	Disagree	Undecided	Agree	Strongly agree	I prefer not to answer
Having sexual relationships is a need that has to be satisfied (such as eating and sleeping)	0□	1□	2□	3□	4□	5□
If I decided not to have sex for the moment, I consider myself prepared to clearly let my partner know it.	0□	1□	2□	3□	4□	5□
In my personal environment I feel pressured to have a partner (to date someone).	0□	1□	2□	3□	4□	5□
In my personal environment I feel pressured to have sex.	0□	1□	2□	3□	4□	5□
It is better to wait until marriage before having sex.	0□	1□	2□	3□	4□	5□
It is OK for youth my age to have sexual relationships just for fun, without commitment.	0□	1□	2□	3□	4□	5□
Males are superior to females	0□	1□	2□	3□	4□	5□
Females are superior to males	0□	1□	2□	3□	4□	5□
In a relationship, what the male says is what should be done.	0□	1□	2□	3□	4□	5□
A woman and a man can perform equally well in any profession	0□	1□	2□	3□	4□	5□

43. Have you ever had sexual relationships? *Remember that by "sexual relationships" we are referring to sex with penetration.*

- 0□ No → Go to next question
- 1□ Yes → Go to question 46
- 2□ I prefer not to answer → Go to question 58

44. If you did not have sexual relationships, we would like to know how important the following reasons were for you.

I did not have sexual relationships because….

	Not important	Slightly important	Moderately important	Important	Very important	I prefer not to answer
I prefer to wait until I meet the person that will share his/her life with me	0□	1□	2□	3□	4□	5□
I do not want to get pregnant or impregnate a girl	0□	1□	2□	3□	4□	5□
I do not want to contract the AIDS virus or any other sexually transmitted infection	0□	1□	2□	3□	4□	5□
I did not find the right person	0□	1□	2□	3□	4□	5□
My partner (s) did not want to have sex	0□	1□	2□	3□	4□	5□
My parents would not agree with this	0□	1□	2□	3□	4□	5□
I think it is not right	0□	1□	2□	3□	4□	5□
I am not economically stable/independent	0□	1□	2□	3□	4□	5□
I am not married	0□	1□	2□	3□	4□	5□
I do not have the right age	0□	1□	2□	3□	4□	5□
I do not feel ready for sex	0□	1□	2□	3□	4□	5□

45. Which of the following best describe your future plans with respect to when to have sexual relationships?

0 ☐ I intend to wait until marriage.
1 ☐ I intend to wait until I commit to someone with whom I would get married sometime after.
2 ☐ I intend to wait until I find someone I love.
3 ☐ I intend to have sex as soon as I have the chance to do so.
4 ☐ I did not think about this yet.
5 ☐ I prefer not to answer.

After any of these responses go to question 58

46. How old were you and your partner during <u>your</u> first sexual relationship?

	10 or less	11	12	13	14	15	16	17	18	19	20 or more	I prefer not to answer
How old were you?	0 ☐	1 ☐	2 ☐	3 ☐	4 ☐	5 ☐	6 ☐	7 ☐	8 ☐	9 ☐	10 ☐	11 ☐
How old was your partner?	0 ☐	1 ☐	2 ☐	3 ☐	4 ☐	5 ☐	6 ☐	7 ☐	8 ☐	9 ☐	10 ☐	11 ☐

47. Did you or your partner use a male or female condom during that first sexual relationship?

0 ☐ No
1 ☐ Yes
2 ☐ I do not remember
3 ☐ I prefer not to answer

48 If you did not use a condom, which was the <u>main reason</u>?

0 ☐ Lack of information (for example: did not know how to use one)
1 ☐ We did not have one available, because they are usually difficult to obtain
2 ☐ We did not have one available at that moment because sex was unplanned.
3 ☐ We did not want to use one (myself, my partner or both)
4 ☐ Another reason
5 ☐ I prefer not to answer

49. Please specify why you did not use a condom during your first sexual relationship?

____

50. <u>In the last 12 months</u>, how frequently did you use condoms during your sexual relationships?

0 ☐ Never
1 ☐ Occasionally
2 ☐ Approximately half of the times
3 ☐ Almost always
4 ☐ Always
5 ☐ I do not remember
6 ☐ I did not have sex in the last 12 months
7 ☐ I prefer not to answer

51. With how many different persons did you have sex in your lifetime?

0□ With only 1 person
1□ With 2 or 3 persons
2□ With 4 or more persons
3□ I prefer not to answer

52. If you have had sexual relationships, we would like to know how important the following reasons were for you.

I had my first sexual relationship because...

	Not important	Slightly important	Moderately important	Important	Very important	I prefer not to answer
I felt a physical urge	0□	1□	2□	3□	4□	5□
I wanted to have fun, to have a good time	0□	1□	2□	3□	4□	5□
Most of my friends already had sex, I did not want to be "different"	0□	1□	2□	3□	4□	5□
I thought it was normal for someone my age to have sex	0□	1□	2□	3□	4□	5□
I wanted to know what it was like, out of curiosity	0□	1□	2□	3□	4□	5□
I wanted to show love to the other person	0□	1□	2□	3□	4□	5□
I wanted to feel accepted, appreciated by the other person	0□	1□	2□	3□	4□	5□
I wanted to keep him/her happy	0□	1□	2□	3□	4□	5□
I wanted to "have" that person	0□	1□	2□	3□	4□	5□
I thought my partner would break up if I said no	0□	1□	2□	3□	4□	5□
My partner told me he/she would break up if I said no	0□	1□	2□	3□	4□	5□
I drank alcohol, smoked marijuana or consumed some other drug	0□	1□	2□	3□	4□	5□
I did not know how to say no to a person that was insisting to have sex	0□	1□	2□	3□	4□	5□
I let myself go during the sexual urge of a moment (kiss, etc.), but having sex was not my initial intention	0□	1□	2□	3□	4□	5□
It was the consequence of having seen sexual images (pictures, videos)	0□	1□	2□	3□	4□	5□

53. When you had sex for the first time, which of the following applied?

0□ I pressured the other person quite a lot
1□ I pressured the other person a little bit
2□ Neither of us pressured each other
3□ The other person pressured me a little bit
4□ The other person pressured me a lot
5□ I don't know/I don't remember
6□ I prefer not to answer

54. Which of the following statements best describes how your first sexual relationship occurred?

0□ It was completely unexpected for me
1□ I expected it to happen soon, but I was not sure of when it would happen
2□ I planned it in advance (but we did not plan it together)
3□ We planned it together in advance
4□ I don't know/I don't remember
5□ I prefer not to answer

55. Concerning your first sexual relationship we want to know how you feel about the timing in your life when it occurred:

0 ☐ I waited too long: I would have preferred to wait LESS.
1 ☐ I think it happened at the right time
2 ☐ It happened too soon: I would have preferred to wait MORE
3 ☐ I don't know
4 ☐ I prefer not to answer

56. In relation to your sexual activity, did you at any moment feel any of these ways?

	Never	Rarely	Sometimes	Frequently	Very frequently	I prefer not to answer
Empty	0 ☐	1 ☐	2 ☐	3 ☐	4 ☐	5 ☐
Used	0 ☐	1 ☐	2 ☐	3 ☐	4 ☐	5 ☐
Disappointed (I expected it to be better)	0 ☐	1 ☐	2 ☐	3 ☐	4 ☐	5 ☐
Dependent, "obsessed" with a person	0 ☐	1 ☐	2 ☐	3 ☐	4 ☐	5 ☐
Dependent, "addicted" to sex	0 ☐	1 ☐	2 ☐	3 ☐	4 ☐	5 ☐

58. In your opinion, when does human life begin?

0 ☐ At fertilization (when the egg and sperm unite)
1 ☐ When the embryo implants in the uterus, approximately 15 days after fertilization
2 ☐ Sometime between embryo implantation in the uterus and birth
3 ☐ At birth
4 ☐ I do not know/I am not sure
5 ☐ I prefer not to answer

59. What is your opinion concerning abortion?

0 ☐ I disagree with abortion
1 ☐ I agree with abortion, but only in certain circumstances
2 ☐ I agree with abortion, whenever the woman decides to have one
3 ☐ I don't know
4 ☐ I prefer not to answer

60. NOW WE WOULD LIKE TO ASK SOME OTHER THINGS ABOUT YOU

61. How many siblings do you have? (Do not include yourself).

0 ❑ None
1 ❑ 1
2 ❑ 2 or more
3 ❑ I prefer not to answer

62. Please indicate with whom you live

	NO	YES	I prefer not to answer
Father	0 ☐	1 ☐	2 ☐
Mother	0 ☐	1 ☐	2 ☐
Another adult (guardian…)	0 ☐	1 ☐	2 ☐
Siblings	0 ☐	1 ☐	2 ☐
Grandparents	0 ☐	1 ☐	2 ☐
Other	0 ☐	1 ☐	2 ☐

63. What is your parents' marital status? If either of them are deceased, please answer with their marital status when both were alive.

0□ They never got married (to each other)
1□ Married
2□ Separated/divorced; neither has remarried or had a stable partner
3□ Separated/divorced; at least one has a stable partner or is remarried
4□ Other
5□ I prefer not to answer

66. Please describe how often the following situations apply to your life.

	Never	Almost never	Sometimes	Almost always	Always	I prefer not to answer
I usually have dinner with my parents	0□	1□	2□	3□	4□	5□
I help with housework	0□	1□	2□	3□	4□	5□
I plan my homework	0□	1□	2□	3□	4□	5□
I do things without thinking	0□	1□	2□	3□	4□	5□
I say things without thinking	0□	1□	2□	3□	4□	5□
I usually finish the things/projects that I start	0□	1□	2□	3□	4□	5□
I usually save money	0□	1□	2□	3□	4□	5□

67. We would like to know to what extent you consider the following relationships satisfactory:

	Not at all	A little	More or less	Somewhat	Very much	Deceased	I prefer not to answer
I consider my parents' relationship (between them) satisfactory	0□	1□	2□	3□	4□	5□	6□
I consider my relationship with my mother satisfactory	0□	1□	2□	3□	4□	5□	6□
I consider my relationship with my father satisfactory	0□	1□	2□	3□	4□	5□	6□

68. In general, your parents:

	Never	Rarely	Sometimes	Almost always	Always	I prefer not to answer
Let you watch any program on TV	0□	1□	2□	3□	4□	5□
Make you feel comforted and supported when you are with them	0□	1□	2□	3□	4□	5□

69. Please indicate how often the following situations apply to your life	Never	Almost never	Sometimes	Almost always	Always	I prefer not to respond
I every now and then like admiring a landscape	0☐	1☐	2☐	3☐	4☐	5☐
I like to question about different things in life	0☐	1☐	2☐	3☐	4☐	5☐
I every now and then like to be in silence without noise or electronic devices	0☐	1☐	2☐	3☐	4☐	5☐
I generally feel free in my life	0☐	1☐	2☐	3☐	4☐	5☐
I feel loved by others (friends, classmates...)	0☐	1☐	2☐	3☐	4☐	5☐
I am generally happy with the life I lead	0☐	1☐	2☐	3☐	4☐	5☐
I can express my thoughts with my friends without fear	0☐	1☐	2☐	3☐	4☐	5☐
At school I feel accepted by my peers	0☐	1☐	2☐	3☐	4☐	5☐
A student at my school has physically or psychologically harmed or assaulted me.	0☐	1☐	2☐	3☐	4☐	5☐
Someone outside of my school has physically or psychologically harmed or assaulted me	0☐	1☐	2☐	3☐	4☐	5☐

70. What religion do you practice or believe in?

0☐ I do not believe in God/I do not know whether God exists ----------------> Go to question 84
1☐ I believe in God but I don´t have any specific religion
2☐ Catholicism
3☐ Protestantism
4☐ Orthodox religion
5☐ Other Cristian Religions
6☐ Islam
7☐ Hinduism
8☐ Buddhism
8☐ Ethnical religion (Africa, Asia, indigenous America, Europe)
9☐ Chinese folk religion (Taoism, Confucianism)
10☐ Shintoism
11☐ Sikhism
12☐ Judaism
13 ☐ Other
14 ☐ I prefer not to answer

71. How often do you do the following:

	Never	Almost never	Occasionally in a year	Occasionally in a month	Once a week	More than once a week	I prefer not to answer
Go to the Church/Temple	0☐	1☐	2☐	3☐	4☐	5☐	6☐
Pray	0☐	1☐	2☐	3☐	4☐	5☐	5☐

72. Do you agree with the following statement? "My faith is an important influence in my life and I am willing to take it into account in my decisions"

	Strongly disagree	Disagree	Neutral	Agree	Strongly agree	I prefer not to answer
Do you agree?	0☐	1☐	2☐	3☐	4☐	5☐

84. Which is the biggest pet you had during your childhood, before 7th grade?
0□ None
1□ Spider or insect
2□ Bunny
3□ Cat
4□ Hamster or Guinea pig
5□ Bird
6□ Dog
7□ Fish, small turtle
8□ Lizard, iguana, other reptile
9□ Other
10□ I prefer not to answer

85. Considering all of the places you went on holiday/vacation during your childhood, before 7th grade, from which one do you have the best memories?
0□ None
1□ Countryside, mountains, village
2□ Camping
3□ Beach
4□ Amusement/theme/water park
5□ Other
6□ I prefer not to answer

86. Which was your least favorite food during your childhood, before 7th grade?
0□ None
1□ Cauliflower or Broccoli or Brussel sprouts
2□ Vegetables in general or fruits
3□ Onion
4□ Brain, liver, kidneys
5□ Fish
6□ Cheese
7□ Mushrooms
8□ Tomatoes or green pees
9□ Other
10□ I prefer not to answer

87. Which of the following sports did you like the most in the 7th grade? If it is not on the list, please choose your next favorite sport.
0□ Basketball
1□ Handball
2□ Horseback riding
3□ Skiing or Snowboarding
4□ Gymnastics, dance or track and field
5□ Golf
6□ Biking
7□ Swimming or water polo
8□ Tennis or squash
9□ Volleyball or baseball
10□ Skating
11□ I don't like any of these sports
12□ I prefer not to answer

88. Which was your favorite color in 7th grade?

0☐ None
1☐ White
2☐ Gray
3☐ Black
4☐ Brown
5☐ Red
6☐ Pink
7☐ Orange
8☐ Yellow
9☐ Green
10☐ Blue
11☐ Violet
12☐ Other
13☐ I prefer not to answer

89. Which was your favorite number in 7th grade?

0☐ None
1☐ 1
2☐ 2
3☐ 3
4☐ 4
5☐ 5
6☐ 6
7☐ 7
8☐ 8
9☐ 9
10☐ 10
11☐ 11
12☐ 12
13☐ 13
14☐ 14
15☐ 15
16☐ 16
17☐ 17
18☐ 18
19☐ 19
20☐ 20
21☐ Other
22☐ I prefer not to answer

91. THANK YOU FOR YOUR WILLINGNESS TO SHARE THIS INFORMATION WITH US.

Lastly, we want you to know that even if we asked whether you have had sexual relationships, and other questions about health-related behaviors, this does not imply that we expected you to have those behaviors. The questions are routinely asked as requirements of the study.

You will finish the survey by clicking this button.
