# Supplementary material for: Project YOURLIFE (What Young People Think and Feel about Relationships, Love, Sexuality, and Related Risk Behavior): Cross-sectional and Longitudinal Protocol
Source: Front Public Health. 2016 Feb 22;4:28. doi: 10.3389/fpubh.2016.00028 (PMC4761899; doi:10.3389/fpubh.2016.00028)
Supplement: Supplementary file 4 [file Image_4.PDF]

## MATCHING VARIABLES

Which is the biggest pet you had during your childhood, before 7th grade?

0 None
1 Spider or insect
2 Bunny
3 Cat
4 Hamster or Guinea pig
5 Bird
6 Dog
7 Fish, small turtle
8 Other
9 I prefer not to answer

Considering all places you went on holiday during your childhood, before 7th grade, from which one do you have best memories?

0 None
1 Countryside, mountains, village
2 Camping
3 Beach
4 Amusement/theme/water park
5 Other
6 I prefer not to answer

Which was your worst food during your childhood, before 7th grade?

0 None
1 Cauliflower / broccoli
2 Vegetables in general
3 Onion
4 Brain, liver, kidneys
5 Fish
6 Cheese
7 Blood sausage
8 Soup
9 Other
10 I prefer not to answer

Which of the following sports you liked the most at 7th grade? If it is not on the list, please choose your next favorite sport.

0 Basketball
1 Handball
2 Horseback riding
3 Skiing
4 Gymnastics or track and field
5 Golf
6 Biking
7 Swimming orwater polo
8 Tennis or squash
9 Volleyball
10 Skating
11 I don’t like any of these sports
12 I prefer not to answer

Which was your favorite color at 7th grade?

0 None
1 White
2 Gray
3 Black
4 Brown
5 Red
6 Pink
7 Orange
8 Yellow
9 Green
10 Blue
11 Violet
12 Other
13 I prefer not to answer

Which was your favorite number at 7th grade?

0 None
1 1
2 2
3 3
4 4
5 5
6 6
7 7
8 8
9 9
10 10
11 11
12 12
13 13
14 14
15 15
16 16
17 17
18 18
19 19
20 20
21 Other
22 Iprefer not to answer
